# An efficient NMR method for the characterisation of ^14^N sites through indirect ^13^C detection

**DOI:** 10.1039/c3cp50787d

**Published:** 2013-04-15

**Authors:** James A. Jarvis, Ibraheem M. Haies, Philip T. F. Williamson, Marina Carravetta

**Affiliations:** a Centre for Biological Sciences , University of Southampton , Highfield Campus , Southampton , SO17 1BJ , UK . Email: P.T.Williamson@soton.ac.uk; b School of Chemistry , University of Southampton , Highfield Campus , Southampton , SO17 1BJ , UK . Email: marina@soton.ac.uk; c College of Science , University of Mosul , Mosul , Iraq

## Abstract

Nitrogen is one of the most abundant elements and plays a key role in the chemistry of biological systems. Despite its widespread distribution, the study of the naturally occurring isotope of nitrogen, ^14^N (99.6%), has been relatively limited as it is a spin-1 nucleus that typically exhibits a large quadrupolar interaction. Accordingly, most studies of nitrogen sites in biomolecules have been performed on samples enriched with ^15^N, limiting the application of NMR to samples which can be isotopically enriched. This precludes the analysis of naturally occurring samples and results in the loss of the wealth of structural and dynamic information that the quadrupolar interaction can provide. Recently, several experimental approaches have been developed to characterize ^14^N sites through their interaction with neighboring ‘spy’ nuclei. Here we describe a novel version of these experiments whereby coherence between the ^14^N site and the spy nucleus is mediated by the application of a moderate rf field to the ^14^N. The resulting ^13^C/^14^N spectra show good sensitivity on natural abundance and labeled materials; whilst the ^14^N lineshapes permit the quantitative analysis of the quadrupolar interaction.

## Abstract 2

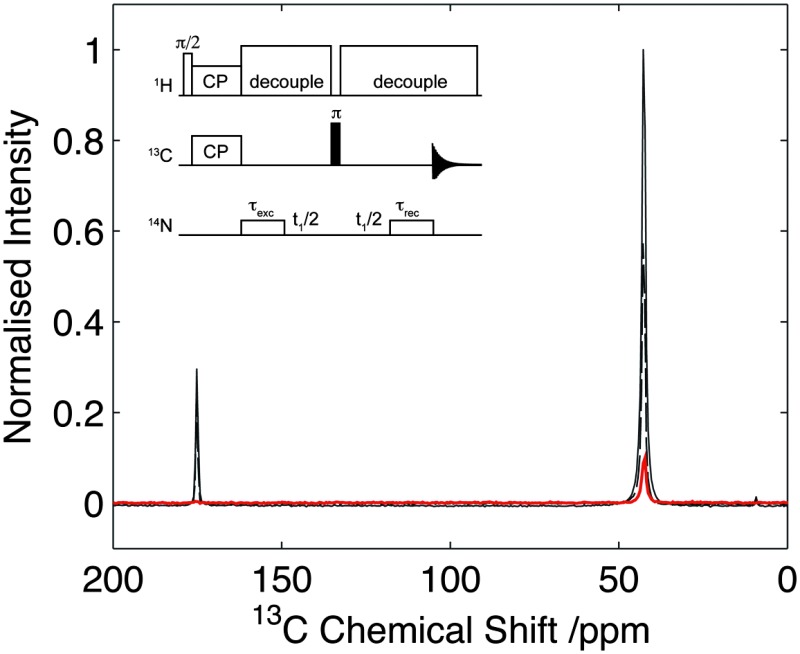
An efficient NMR methods for the characterisation of ^14^N sites has been developed with efficiencies suitable for the quantitative analysis of biomolecular and natural abundance systems.

## Introduction

Nitrogen is an abundant element in (bio)molecular systems playing important structural and functional roles in both proteins and nucleic acids. Accordingly, methods for the analysis of the naturally abundant isotope of nitrogen, ^14^N, by nuclear magnetic resonance (NMR) are clearly desirable. With its high natural abundance, 99.63% and a gyromagnetic ratio 71% of the widely exploited isotope ^15^N, ^14^N would appear to be an attractive nucleus for investigation by NMR.

However, ^14^N has remained largely unutilized due to the fact that it is a spin-1 nucleus that typically exhibits a moderately large quadrupolar interaction, often a few MHz in size, making its direct detection challenging. However, the presence of the large ^14^N quadrupolar interaction can also be advantageous providing valuable information regarding the conformation and dynamics. Its accurate and efficient characterisation by NMR can potentially lead to novel insights into the structure and dynamics of (bio)molecular systems.

A number of methods have been developed to study ^14^N by NMR. Direct detection has been employed for sites with small quadrupolar interactions^1–4^ or single crystal studies.^5^ For ^14^N sites with quadrupolar interactions up to 1 MHz in size, adiabatic excitation schemes have proved effective at exciting the broad spectra.^6^ However the analysis of larger quadrupolar interactions has typically relied on the “piece-wise” acquisition of a series of sub-spectra with different transmitter offsets, which are subsequently recombined to produce the overall powder pattern.^7^ Such approaches have found application under static and magic-angle spinning (MAS) conditions. More recently however a class of MAS experiments has been proposed which utilizes a proximal spy nucleus, typically ^13^C or protons, to probe the spectral properties of an adjacent ^14^N site.^8–22^

Experiments utilizing these ‘spy’ nuclei approach lead to the indirect detection of the ^14^N signal and are loosely based on the analogous liquid-state HMQC and HSQC experiments. However in this instance, coherence between the ^14^N and the spy nuclei is mediated not only by the J-coupling but also by the second-order quadrupolar–dipolar interactions, sometimes referred to as the residual dipolar splittings, which are not completely averaged by MAS,^23^ and by the dipole–dipole interaction if suitable recoupling methods are adopted.^11,14,21^ These methods have enabled the spectral properties of the ^14^N site to be determined without the need to uniformly excite or detect the ^14^N spectrum. To date, they have proved useful in the structural and dynamic analysis of small molecules.^24,25^

**Fig. 1:**
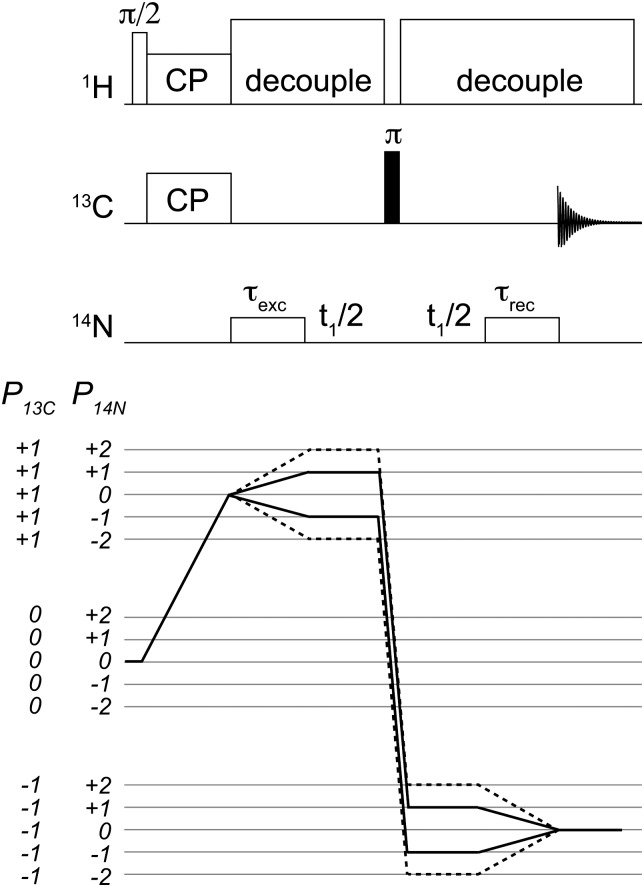
Pulse sequence used for the acquisition of 2D ^14^N/^13^C correlation spectra. Coherence selection pathways are shown for the single quantum (solid line) and double quantum (dashed-line). Analogous 1D spectra were acquired with the same sequence with no *t*_1_ evolution. Conditions for cross-polarization, decoupling and ^14^N pulses are as described in the text.

Here we report on an analogous class of experiments that utilizes a proximal spy nucleus, specifically ^13^C, to observe the spectral properties of the ^14^N sites. Earlier methods typically generated coherence between the ^14^N and the spy nuclei in two steps: first, the second-order quadrupolar–dipolar interactions was allowed to evolve freely during a period of mixing; then a ^14^N pulse of duration of tens of microseconds was applied. In contrast the experiment described here generates coherence between the ^14^N and spy nuclei under the action of a long, continuous, moderate (10's kHz) rf field applied to the ^14^N at or close to its Larmor frequency, with no periods of free evolution (see Fig. 1). We have applied this experiment to a number of systems possessing a range of quadrupolar interactions up to about 3 MHz in size, sizes that are commonly found in organic molecules. Experimentally, by generating the ^14^N/spy nuclei coherence under the application of a ^14^N field, the resulting pulse sequence proves to be very robust, giving good efficiencies across a range of compounds with next to no optimization. It is well compensated for small changes in pulse duration, rf amplitude and resonance offset, making it easy to implement and apply. Indeed, experimental efficiencies are sufficient to permit the analysis of materials containing only natural abundance ^14^N and ^13^C in a matter of hours.

Furthermore, good agreement is observed between experimental data and numerical simulation, allowing for the accurate characterisation of the quadrupolar interaction at the ^14^N site and providing a novel route to structural and dynamic information in small molecule, materials and biomolecular systems.

## Theoretical description

The pulse sequence used in this work is shown in Fig. 1. After the initial step of ramped cross-polarization^26,27^ (CP), the transverse ^13^C magnetization evolves under the effect of the ^13^C–^14^N interactions during the rotor-synchronised ^14^N pulse, of duration *τ*_N_. During *τ*_N_, the spin dynamics is influenced by the rf field, J-couplings, through-space dipolar couplings, quadrupole interaction and higher-second quadrupole–dipole terms.^10,19,23^ The result is a complicated density operator. It has been verified by means of numerical simulations using SPINEVOLUTION^28^ that both ^14^N single quantum (*T*N1±1 and *T*N2±1) and double quantum (*T*N2±2) terms are generated with efficiencies well in excess of 20% with respect to the initial equilibrium magnetization. Hence this sequence can be effectively used to record both HSQC and HMQC-type of ^14^N–^13^C correlation experiments.

The pulse sequence presented for indirect ^14^N leads to the generation of many different coherence orders, a property that is not unique to this scheme.^10,12,13,15–17^ In the studies presented, the coherence order of interest has been isolated purely by means of phase cycling, as shown in Fig. 1. In this work, we demonstrate experimental results for ^14^N SQ measurements.

Following a strong π pulse on ^13^C, the signal is reconverted into observable ^13^C magnetization during another ^14^N pulse of equal duration, *τ*_N_. Omission of the delays between the two ^14^N pulses and the π-pulse allows the acquisition of ^14^N-filtered ^13^C spectra. Alternatively, an incremental delay can be inserted between the two ^14^N pulses and the π pulse in order to allow the ^14^N coherences to evolve, leading to the acquisition of two-dimensional (2D) spectra for indirect ^14^N detection. The time increment in the indirect dimension must be chosen to be an integer multiple of the rotor period, *i.e.*, Δ*t*_1_ = *nτ*_r_, in order to ensure effective averaging of the first order quadrupolar interaction.

The π pulse has the purpose of refocusing ^13^C isotropic and anisotropic ^13^C chemical shifts during the ^14^N signal evolution in the indirect dimension.

## Experimental section

### Materials

Natural abundance and ^13^C labelled samples were purchased from Sigma and CIL (MA, USA) respectively and used without further purification. All samples used in this work were used as purchased without further purification. Gly-(1,2-^13^C_2_)Gly-Gly (>95%) was synthesized by Peptide and Protein Research using FMOC-(1,2-^13^C_2_)Gly purchased from CIL (MA, USA). The peptide was lyophilized and used without further purification for the studies conducted.

### Numerical simulations

All 1D and 2D simulations have been performed using the SPINEVOLUTION-3.4.3 software package, whose capabilities have recently been extended to deal with quadrupolar nuclei.^28^ For the sake of simplicity, the effect of protons is neglected in our simulations, which include only one ^13^C–^14^N spin pair. Specific details of the parameters used for each set of simulations are given in Table 1. The simulations are performed using 6044 powder points with the ZCW 3D angle sets.^29^ It was verified that the simulated spectra converged under these conditions and there was no need for larger angle sets when dealing with large quadrupolar interactions (*e.g.* amide, 3.01 MHz). For the simulations, two limiting cases were considered, with quite different quadrupole size and orientations: for an NH_3_ group, as found in glycine, a quadrupole interaction of 1.18 MHz, *η* = 0.54 aligned collinear with the dipolar interaction was employed.^30^ For amide groups, as in the case of Gly-(1,2-^13^C_2_)Gly-Gly, we used 3.01 MHz, *η* = 0.48 with a relative dipole–quadrupole orientation of (0°,90°,120°), as described in Rabbani *et al.* 1987^31^ and Bak *et al.* 2002.^32^

**Table 1:** Parameters used in numerical simulations

Simulation	*C*_Q_/MHz	*η*_Q_	Dipolar coupling/kHz	Quadrupolar tensor orientation wrt dipolar tensor (*α*,*β*,*γ*)
Glycine (amine)^30^	1.18	0.54	–0.716	(0°,0°,0°)
Triglycine-N2 (amide)^31,32^	3.01	0.48	–0.928	(0°,90°,120°)

### NMR experiments

Experimental data were acquired on a Bruker Avance-II spectrometer operating at 14.1 T (^1^H, ^13^C and ^14^N Larmor frequencies of 600, 150 and 43.5 MHz respectively) equipped with a Bruker 2.5 mm triple resonance MAS probe modified in house to tune to ^1^H, ^13^C and ^14^N. ^13^C magnetization was generated by means of a CP using a 95 to 105% linear ramp of the proton field optimised to match a ^13^C spin-lock field of 50 kHz. The rf amplitudes were calibrated on crystalline ammonium chloride. All ^14^N spectra are referenced to crystalline ammonium chloride with a single resonance at 35.9 ppm.^33^

Excitation and reconversion ^14^N pulses of equal duration were applied with a nutation frequency of 35 kHz (glycine and histidine) or 50 kHz (Gly-(1,2-^13^C_2_)Gly-Gly). Typically the ^14^N pulse lengths, *τ*_N_, were 2 ms although in each case this was optimised to give optimal efficiency and further details are given in the respective figure legends. During the ^14^N pulses protons were decoupled using SPINAL64 decoupling with an rf amplitude of 115 kHz. Effective heteronuclear decoupling during the long ^14^N pulses is very critical to achieve good experimental efficiency with this approach. During the *t*_1_ and *t*_2_ intervals, protons were decoupled using SPINAL64 with a reduced rf amplitude of 100 kHz to mitigate sample heating.^34^

^13^C/^14^N 2D correlation spectra were acquired phase sensitively using States acquisition.^35^ Unless stated, data were processed with 30 Hz line-broadening in the *t*_2_ prior to Fourier transform and the data zero filled to 1024 data points in each dimension. In the indirect dimension the data was acquired rotor synchronously with Δ*t*_1_ = *τ*_r_, until the signal had decayed to noise, typically no more than 64 *t*_1_ increments.

## Results and discussion

When compared to other methods reported in the literature, this ^14^N excitation scheme is efficient and very easy to set-up, as the parameters vary little among different spin systems and any variations are typically minor and can to a large extent be predicted *a priori*, *i.e*., stronger rf pulses on ^14^N on amide groups. If all power levels are properly optimised on a model sample, like NH_4_Cl, the sequence can be run on the sample of interest with minor to no optimization. These properties are highlighted in the following examples given below.

### Glycine

**Fig. 2:**
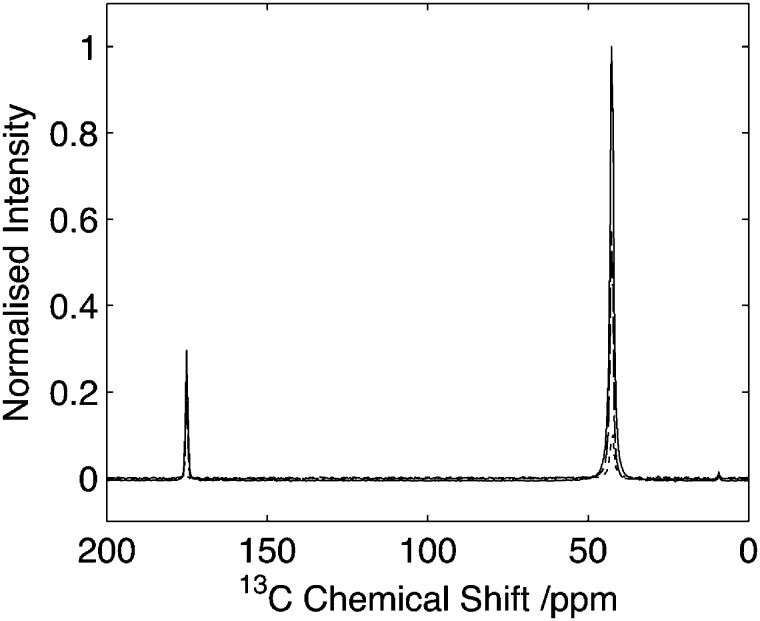
Comparison of HNC efficiency on U-^13^C_2_-glycine. Cross polarization spectrum (solid line), echo spectrum (dashed) in comparison to optimal transfer through ^14^N (dotted). Intensity of the C_α_ signal of glycine is 9% with respect to the CP signal. Data acquired with 25 kHz spinning. Data processed with 30 Hz line-broadening prior to Fourier transform.

Initial studies were performed on U-^13^C_2_-glycine to assess the efficiency of the experiment. Comparison of the ^14^N/^13^C filtered experiment conducted at 25 kHz MAS with a 2 ms ^14^N pulse of 35 kHz rf amplitude resulted in a signal from the C_α_ site which was 10% of that observed from direct CP (Fig. 2). When comparison is made with the equivalent echo experiment with a 2 ms evolution period before and after the refocusing pulse, this figure increases to 17%. We attribute the improved efficiency to the unfavourable *T*_2_ relaxation and evolution of ^13^C–^13^C J-couplings that occurs during the echo period which together significantly attenuates the ^13^C signal intensity. These points are discussed in more detail below.

**Fig. 3:**
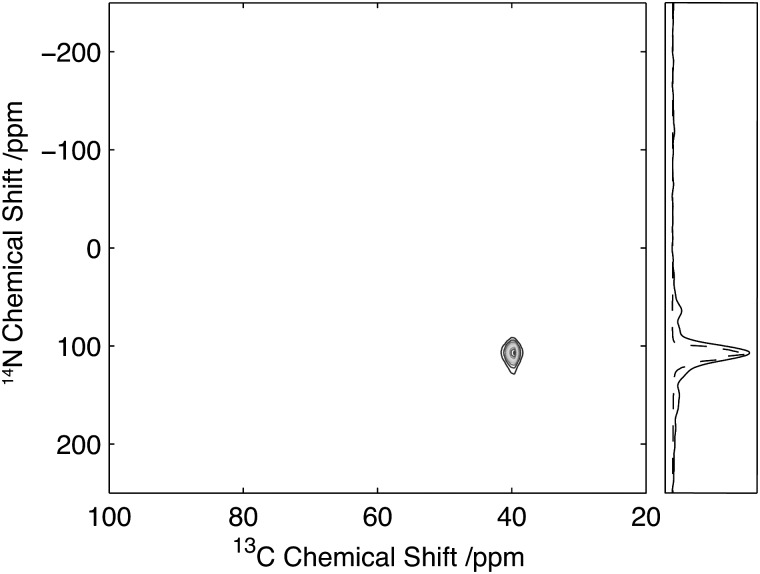
^13^C/^14^N 2D correlation spectrum of U-^13^C_2_-glycine. Data acquired with 25 kHz MAS and 2 ms, 35 kHz ^14^N excitation and reconversion pulses. Slice through the C_α_ glycine peak (solid) with the corresponding simulated spectrum (dashed).

The corresponding 2D ^13^C/^14^N correlation spectrum of U-^13^C_2_-glycine is shown in Fig. 3 with a strong correlation observed between the amine and the C_α_ of the glycine. Comparison of the ^14^N slice with that obtained by numerical simulations with literature parameters,^30^ which describe the quadrupolar interaction to be 1.18 MHz with an asymmetry of *η* = 0.54 and aligned with the ^13^C–^14^N dipolar interaction, shows good agreement between them. This suggests that for smaller quadrupolar interactions, the lineshapes obtained from this experiment can be used to quantitatively characterise the quadrupolar interaction at the ^14^N site.

**Fig. 4:**
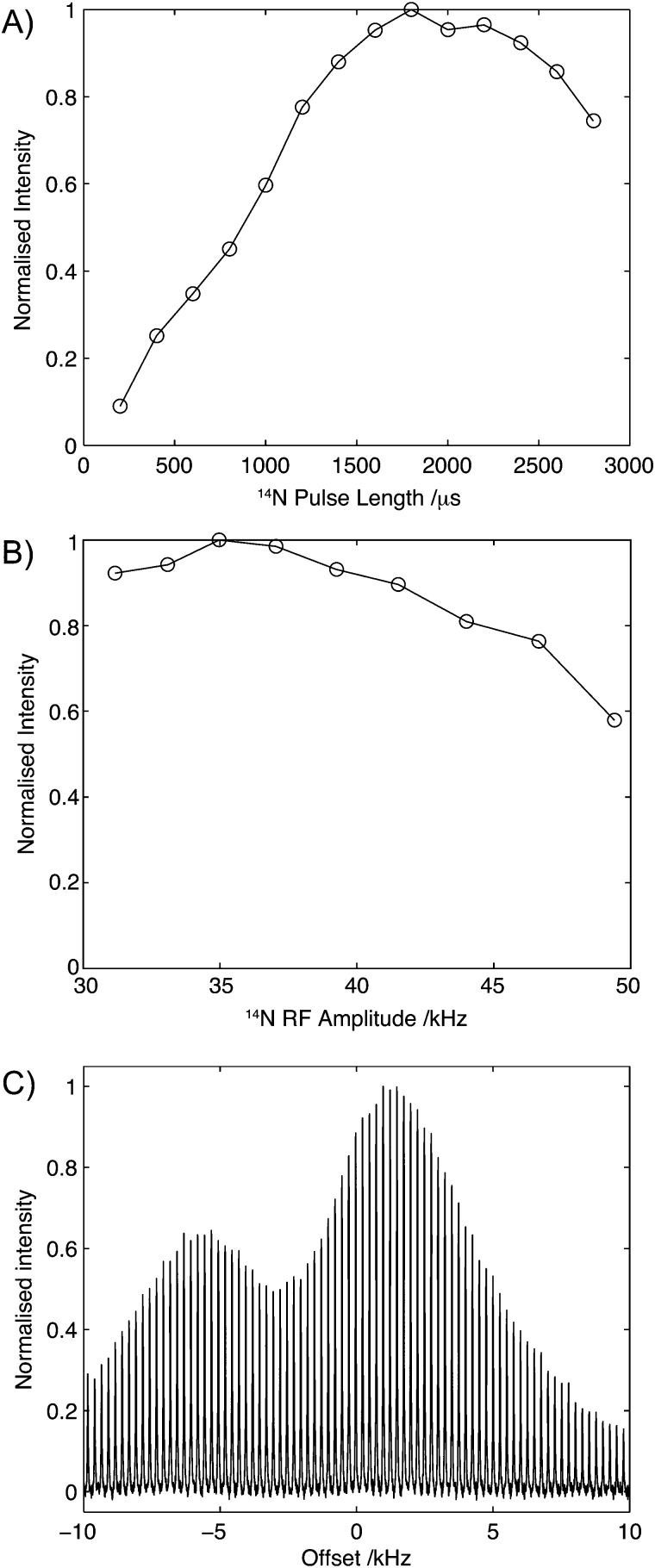
Effect of pulse length (A), rf amplitude (B) and ^14^N offset (C). In each case, the data was acquired with all other parameters optimised. In each case data normalised such that the maximum intensity is 1.0.

To assess the robustness of this experiment, the efficiency of transfer was measured as a function of ^14^N pulse duration, amplitude and resonance offset on U-^13^C_2_-glycine. The results of these studies are shown in Fig. 4. Fig. 4A shows the build-up of the C_α_ intensity as a function of the ^14^N pulse duration, *τ*_N_, for a fixed field strength of 35 kHz with a broad maximum between 1800 μs and 2200 μs. The broad maximum observed here remained remarkably similar across all compounds reported in this manuscript, with little variation between compounds exhibiting large (∼3 MHz) and small (∼1 MHz) couplings. Experimentally, we typically observed that during the experimental setup the pulse length can be set to 2 ms, with moderate improvements (∼5%) in sensitivity being realised through subsequent experimental optimisation. Fig. 4B shows the effect of ^14^N rf amplitude on the C_α_ intensity. The experimental efficiencies observed in ^14^N filtered ^13^C experiments conducted on U-^13^C_2_-glycine show a broad maximum centred at 35 kHz. Beyond this, a drop in efficiency was observed, suggesting that for quadrupolar couplings of about 1 MHz, optimal transfer between the ^13^C and ^14^N can be attained with currently available MAS probes. Furthermore, the weak dependency of experimental efficiency on the strength of the rf amplitude close to the optimal field makes the implementation of the experiment less challenging.

Due to the width of the ^14^N spectrum, a major consideration in the implementation of these experiments is the dependence of the efficiency with respect to the ^14^N offset. During the setup of the experiments, the initial ^14^N offset was set to that measured for the ^14^N signal of ammonium chloride. The efficiency of the experiment a function of the ^14^N offset is shown in Fig. 4C and similar profiles were observed for the other compounds studied. Importantly and in contrast to direct detection, optimal efficiencies are observed close to the isotropic chemical shift values, which are frequently known from studies of other model compounds or through comparison with the respective chemical shifts observed in ^15^N spectra.^36,37^ Over the range of offsets studied here, two peaks in efficiency are observed. However, within 10 kHz (∼250 ppm) of the isotropic chemical shift of ammonium chloride, efficiencies of over 50% are recorded. The relatively high efficiencies observed across a range of frequencies which match the distribution of ^14^N chemical shifts in most organic molecules again serves to simplify the implementation of this experiment.

**Fig. 5:**
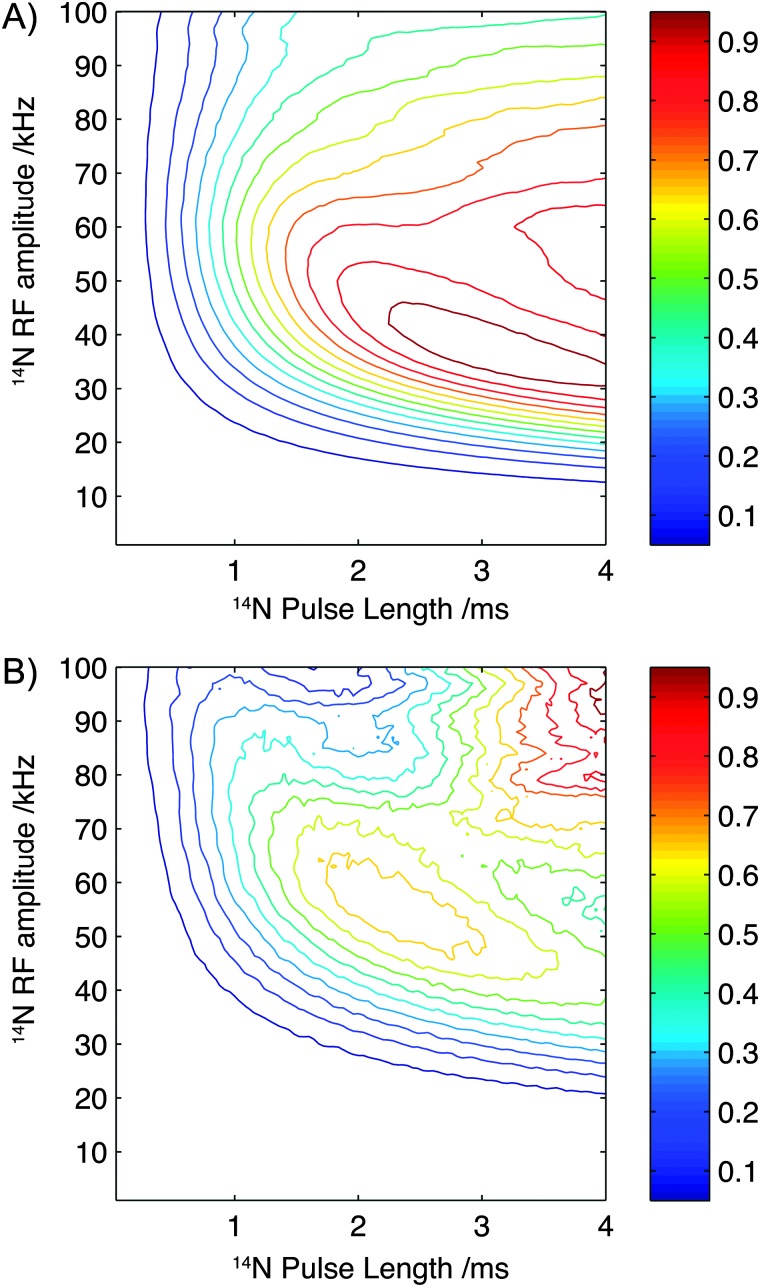
Contour plots showing the simulated efficiency of the experiment as a function of pulse length and rf amplitude for parameters describing the structure and spin interactions present in glycine (A) and Gly-(1,2-^13^C)Gly-Gly (B). Each plot is scaled such that the maximum intensity is normalised to 1.0.

To further investigate the sensitivity of the experiment to pulse length and field strength, numerical simulations have been performed with parameters which reflect the structure and spin interactions present in glycine and a single ^13^C–^14^N spin pair, while neglecting the presence of protons and relaxation effects. The results of these simulations are plotted in Fig. 5A. In agreement with experimental observations, optimal efficiencies are observed at experimentally accessible rf field strengths of approximately 40 kHz with efficiencies plateauing when pulse lengths exceed 2 ms.

### Application to biopolymers

**Fig. 6:**
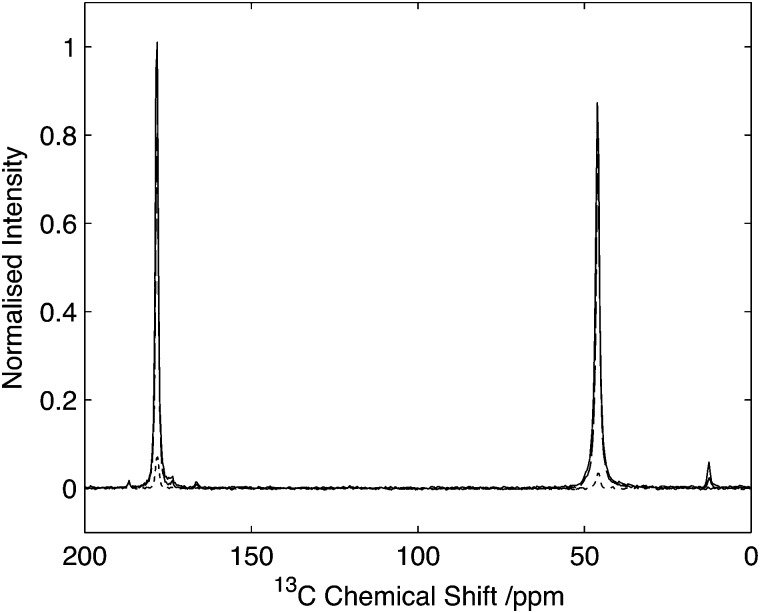
Comparison of HNC efficiency on Gly-(1,2-^13^C_2_)Gly-Gly. Cross polarization spectrum (solid line), echo spectrum (dashed) in comparison to optimal transfer through ^14^N (dotted). Intensity normalised with respect to maximal CP signal. Data acquired with 25 kHz spinning. Data processed with 30 Hz line-broadening prior to Fourier transform.

Results on model amino acids revealed a robust and efficient experiment, however the quadrupolar interaction typically observed in these compounds is relatively small (∼1.18 MHz)^30^ due to the high degree of symmetry and rotation of the amine group. Furthermore the quadrupolar interaction is typically aligned with the N–C bond which may further influence transfer efficiencies. To ascertain the effectiveness of this experimental strategy on compounds with significantly larger quadrupolar couplings and with geometries mimicking those found in biomolecules, experiments have been conducted on Gly-(1,2-^13^C_2_)Gly-Gly where the labelled C*i*α and CO^*i*^ sites in residue *i* = 2 are found adjacent to the nitrogens at residues *i* = 2 and *i* = 3 respectively. In this tripeptide, the ^14^N in residues *i* = 2 and *i* = 3 are contained within the peptide bond, exhibiting an sp^2^ hybridization state with a lower symmetry than the amines studied previously, resulting in a quadrupolar interaction of 3.01 MHz.^31^ A comparison of the ^14^N filtered spectrum compared to the direct CP and the corresponding echo is shown in Fig. 6. For the carbonyl resonance (178.2 ppm) efficiencies of 7% are observed when compared with direct CP rising to 9% when compared to the corresponding echo experiment. In contrast, the C_α_ resonance (45.5 ppm) exhibits efficiencies of 4% when compared with the direct CP rising to 4.7% for the corresponding echo experiment. These efficiencies are comparable with efficiencies reported by other authors for indirect detection of ^14^N in when applied to systems with similar quadrupolar interactions.^8–17,19–21^

To assess the sensitivity of the experiment to rf amplitude and ^14^N pulse lengths when applied to polypeptides, numerical simulations were performed. In this instance, using parameters that mimic the chemical structure and spin interactions found in the backbone of a polypeptide chain, and perhaps most importantly the larger quadrupolar interaction (3.01 MHz).^31^ The results of these simulations are plotted in Fig. 5B. As observed for the simulation of glycine, above 2.0 ms the efficiency shows only a weak dependency on pulse length although an optimal performance is observed between 2.0 and 2.5 ms. In contrast to the simulations calculated using parameters which represent glycine the optimal efficiency is now observed at a slightly higher ^14^N rf amplitude, with an experimentally accessible maximum observed at fields of 50–55 kHz. Interestingly Fig. 5B highlights that for larger quadrupolar interactions, better theoretical efficiencies may be expected at higher field strength (100 kHz) and with slightly longer pulse lengths, indicating that improvements in hardware and dedicated ^14^N probes may further enhance the efficiencies of this experiment quite significantly.

**Fig. 7:**
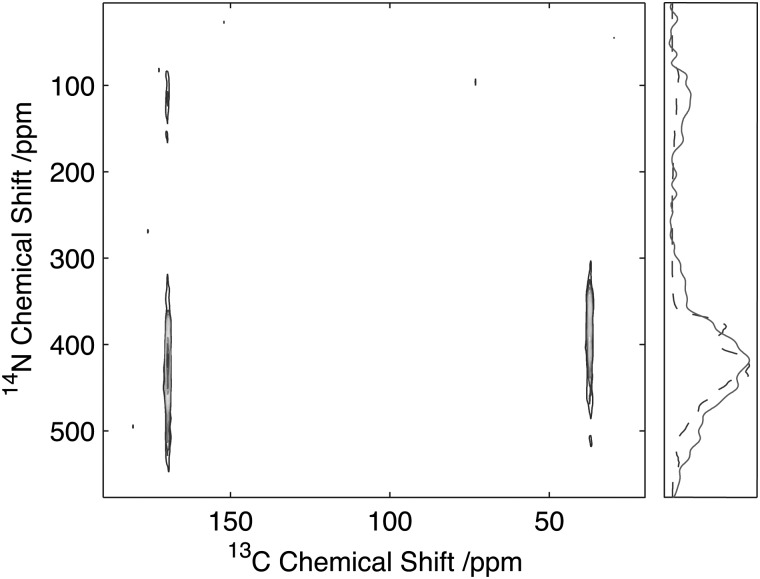
^13^C/^14^N 2D correlation spectrum of Gly(1,2-^13^C_2_-Gly)Gly. Data acquired with 25 kHz MAS and 2 ms, 35 kHz ^14^N excitation and reconversion pulses. Slice through the CO Gly_2_ resonance (solid) with the corresponding simulated spectrum (dashed).

The 2D ^14^N/^13^C correlation spectra for Gly-(1,2-^13^C)Gly-Gly is shown in Fig. 7. Analysis of the resonance between the CO-Gly_2_ (178.2 ppm, ^13^C) and the N-Gly_3_ reveals a strong resonance centred at 420 ppm in the ^14^N dimension with intensity spread over approximately 280 ppm (see slice in Fig. 6). In addition, a weaker resonance is observed at 120 ppm with intensity spread over approximately 100 ppm (see slice in Fig. 6). The resonance between N-Gly_2_ and the C_α_-Gly_2_ (45.5 ppm, ^13^C) shows a single resonance peaking at 350 ppm with the ^14^N with intensity spread over 165 ppm. Direct comparison of the ^14^N slice through the carbonyl resonance with numerical simulations revealed a relatively poor agreement with the experimental data, with the simulated spectra showing a number of well-defined features that were poorly characterised in the experimental data. A better fit of the experimental data was obtained when the simulations were performed when the magic-angle deviated from 54.74° by 0.05°. This highlights the importance of setting the magic-angle accurately when the ^14^N lineshapes are to be studied quantitatively and recently a number of protocols have been proposed which offer improvements in accuracy over utilizing the sidebands in the ^79^Br spectra of KBr when setting the magic angle.^38,39^ Despite the ^14^N spectra being significantly broader than those observed for the equivalent amine, these spectra demonstrate that for quadrupolar couplings up to ∼3.0 MHz, spectra can be acquired with reasonable efficiency with a quality sufficient to enable the characterisation of the quadrupolar interactions at the individual sites.

### Application to natural abundance materials

**Fig. 8:**
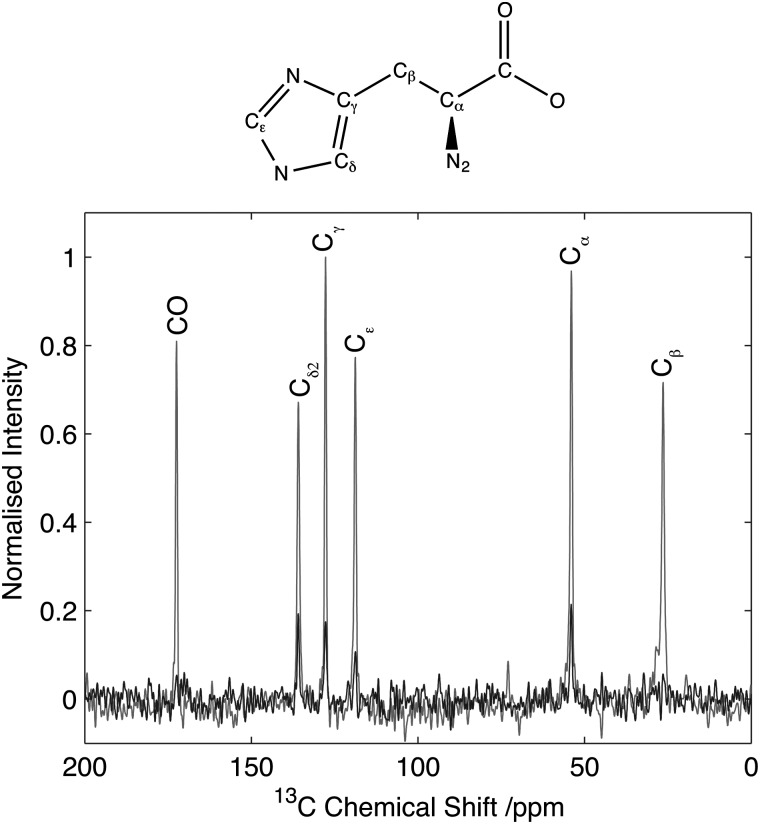
Comparison of HNC efficiency on natural abundance histidine. CP spectrum (grey) in comparison to optimal transfer through ^14^N (black). Data normalized to maximum CP intensity. Intensity of the C_α_ signal of histidine is 22% with respect to the CP signal. Data acquired with 15 kHz spinning. Data processed with 30 Hz line-broadening prior to Fourier transform.

**Fig. 9:**
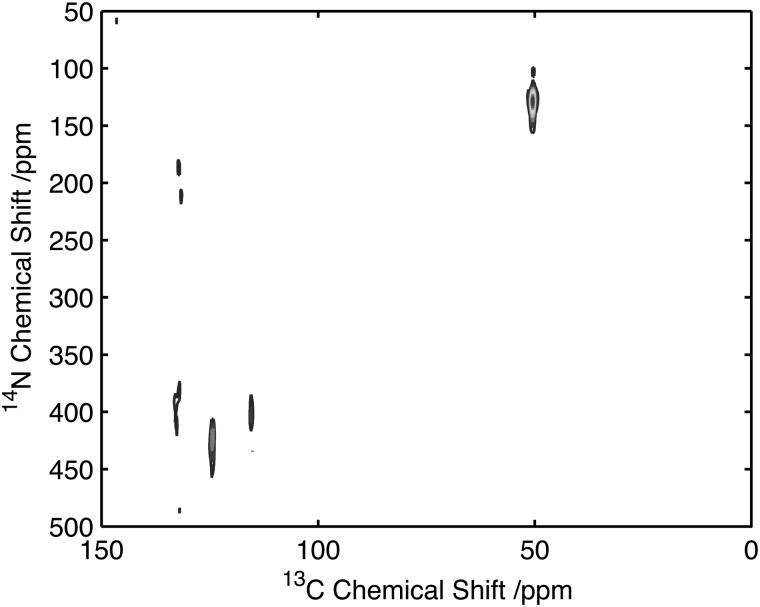
^14^N/^13^C correlation spectra of natural abundance histidine acquired on 12 mg of material in 20 h. Data acquired with 25 kHz MAS and 2 ms, 35 kHz ^14^N excitation and reconversion pulses.

To assess the feasibility of using this method to study samples without ^13^C isotope enrichment that would facilitate the broader application of the experiment to unlabelled biomaterials and environmental samples spectra were acquired of natural abundance l-histidine. Spectra of l-histidine recorded with 15 kHz spinning and a 2 ms ^14^N pulse with a field strength of 35 kHz showed excellent sensitivity compared to labelled samples with efficiencies of between 16 and 23% depending on the carbon site when compared to the direct CP. (Fig. 8). The spectrum ^14^N/^13^C filtered spectrum of histidine also serves to highlight the broadbanded nature of the experiment with a range of different chemistries across a range of ^13^C resonances frequencies all showing relatively high levels of efficiencies. The efficiencies observed were sufficient to permit the acquisition of 2D data set in less than 20 h on 12 mg of material (Fig. 9). The presence of the ^13^C spin echo means that this method will be inherently more sensitive (when compared to direct ^13^C CP) when the *T*_2_ of the sample is long. As demonstrated in the case of the natural abundance histidine, this serves to highlight the importance of efficient decoupling during this experiment. As postulated from our studies on U-^13^C_2_-Glycine, significant enhancements in efficiency are observed in this natural abundance material as the unfavourable evolution of the homonuclear ^13^C J-couplings that would otherwise attenuate the overall echo intensity are absent. Such an observation suggests that the sensitivity of this method when applied to labelled materials would be significantly enhanced through suppression of the homo-nuclear J-couplings, with selective refocusing schemes potentially offering significant improvement for the study of backbone amide sites.^40^

## Conclusion

Here we report on a novel method for studying ^14^N sites indirectly through proximal spy nuclei. In contrast to earlier methods, transfer is mediated through extended (ms) periods of rf irradiation at moderate field strength (30–50 kHz). Experimental studies of a range of compounds supported with extensive numerical simulations have demonstrated the experiment to be robust, with little variation in efficiency across a relatively broad range of pulse lengths and rf amplitudes. Furthermore, the relatively moderate demands in rf levels and efficiencies showing only a moderate dependence on the structure and nuclear spin interactions present within the sample the experiment has proven simple to implement. Two-dimensional spectra were obtained for a range of ^14^N sites and comparison of the simulated lineshapes shows good agreement, allowing the characterization of the ^14^N quadrupolar interaction for all the organic compounds considered here. As highlighted above, for particular molecular systems significant improvement can be envisaged be it through the suppression of homonuclear J-couplings in labelled molecules or through the application of alternative ^14^N excitation schemes.^22^

The ease of implementation and the good experimental efficiencies observed offer the potential to characterise the ^14^N sites in a range of labelled and unlabelled materials providing novel routes to the characterization of their molecular structure and dynamics. These properties provide opportunities to study nitrogen in sites where before our inability to introduce isotope labels has hindered studies, areas including environmental samples, pharmaceuticals and other natural products.
